# Antibody response to SARS-CoV-2 natural and breakthrough infection in patients undergoing maintenance hemodialysis: A prospective cohort study over 4 months

**DOI:** 10.1016/j.heliyon.2024.e38545

**Published:** 2024-09-26

**Authors:** Yingji Chen, Xiaming Zhang, Mi Zhou, Ping Wu, Juzhen Yan, Chen Sun, Yinghong Zhang, Xiaoyin Zheng

**Affiliations:** a Department of Nephrology, The Affiliated Hospital of Hangzhou Normal University, Hangzhou, China; b Department of Clinical Laboratory, The Affiliated Hospital of Hangzhou Normal University, Hangzhou, China

**Keywords:** Hemodialysis, SARS-CoV-2, IgG, Natural infection, Breakthrough infection

## Abstract

**Background:**

Patients undergoing maintenance hemodialysis (MHD) are susceptible to severe acute respiratory syndrome coronavirus 2 (SARS-CoV-2) infection. However, the antibody response of these patients to natural and breakthrough infections remains poorly understood.

**Methods:**

Between January 15, 2023, and February 15, 2023, a total of 53 patients undergoing MHD and diagnosed with SARS-CoV-2 infection at The Affiliated Hospital of Hangzhou Normal University were enrolled. They were categorized into the natural (n = 40) and breakthrough infection groups (n = 13) based on their vaccination status before infection. Comprehensive data, including basic clinical information, vaccination status, and routine post-infection blood parameters, were collected from all participants. Blood specimens were drawn monthly after infection, and SARS-CoV-2 receptor-binding domain (RBD) immunoglobulin (Ig) G and IgM tests were conducted. The study included continuous follow-up over 4 months.

**Results:**

During the acute phase of infection, the SARS-CoV-2 RBD IgM positivity was 5 % in patients undergoing MHD who were naturally infected and 15.4 % in those with breakthrough infections. Four months after infection, SARS-CoV-2 RBD IgG positivity in patients with natural and breakthrough infection was 77.5 % and 100 %, respectively. Patients undergoing MHD with breakthrough infection exhibited higher SARS-CoV-2 RBD IgG titers (751.21 signal-to cutoff ratio, S/CO [interquartile range, IQR, 30.54–1173.63]) than those with natural infections (3.43 S/CO [IQR, 1.12–15.6]) (p < 0.0001). No significant differences were observed in SARS-CoV-2 RBD IgG between males and females or among those aged <70 and ≥70 years. Patients who received three doses of the inactivated vaccine produced significantly higher SARS-CoV-2 RBD IgG levels after infection than those who received one or two doses; these differences were significant.

**Conclusions:**

Although patients undergoing MHD exhibit a low rate of SARS-CoV-2 RBD IgM positivity following infection, those vaccinated with inactivated vaccines can generate elevated SARS-CoV-2 RBD IgG levels, particularly those who receive three doses.

## 1 Introduction

Coronavirus disease 2019 (COVID-19), caused by severe acute respiratory syndrome coronavirus 2 (SARS-CoV-2), has emerged as a global pandemic [1]. Among the most vulnerable are patients undergoing maintenance hemodialysis (MHD), who face a considerably elevated risk of infection compared with the general population because of the required thrice-weekly hospital visits for life-sustaining treatment [2]. A British study revealed considerably higher COVID-19-related death rates (per 10,000) among older residents (29.7) and patients undergoing kidney dialysis (80) than in the primary study population (6.0) [3]. A follow-up study of 3285 patients undergoing hemodialysis (HD) and infected with SARS-CoV-2 reported a 28-day post-infection mortality rate of up to 20 % [4]. Additionally, given that most patients undergoing HD are older and immunocompromised, there is a heightened concern about their susceptibility to severe outcomes [5,6].

Previous studies have focused on the humoral immune response in patients undergoing HD who have been vaccinated against SARS-CoV-2. Current evidence suggests that these patients can produce immunoglobulin (IgG) antibodies, although their seroconversion rates and antibody levels are often lower than those in the general population [[7], [8], [9], [10]]. Little is known about the antibody response and duration in patients undergoing MHD after SARS-CoV-2 natural and breakthrough infections. To address these knowledge gaps, we conducted a prospective cohort study to assess the antibody responses in patients undergoing HD after natural and breakthrough infections.

## 2 Materials and methods

### 2.1 Cohort and context

In January 2023, the HD unit at The Affiliated Hospital of Hangzhou Normal University was directly affected by the COVID-19 outbreak. At the time, 145 patients undergoing MHD were actively receiving treatment at our facility. At the entrance, a check-in process was established to welcome patients individually, conduct temperature checks using a temperature gun, and inquire about their health statuses. Patients on HD with a fever or other upper respiratory tract infection symptoms underwent polymerase chain reaction (PCR), routine blood tests, and anti-SARS-CoV-2 antibody assessments. Dialysis for PCR-positive patients was organized into designated zones and time slots to prevent cross-infection, and masks were consistently worn throughout the dialysis period.

This prospective cohort study enrolled patients undergoing MHD with confirmed SARS-CoV-2 infection through nose and throat swab PCR at The Affiliated Hospital of Hangzhou Normal University between January 15, 2023, and February 15, 2023. All participants were 18 years or older, had a dialysis vintage of more than 3 months, and had no history of COVID-19. Based on their vaccination status, the patients were classified into two groups: natural infection and breakthrough infection.

### 2.2 Clinical parameters and sampling

On March 15, 2023, we gathered various clinical data, including post-infection blood cell counts, dialysis vintage, and anti-SARS-CoV-2 antibodies, from participants using the hospital's medical record network system. By reviewing personal health records, we obtained information on the participants' vaccination status, type of vaccine received, and number of doses administered. These data served as the first follow-up (Visit 1). All participants consented to peripheral blood sampling, and their anti-SARS-CoV-2 antibodies were assessed at four follow-up visits (Visits 2–5) at months 1, 2, 3, and 4 post-infection.

### 2.3 Serological assays for detecting anti-SARS-CoV-2 antibodies

Venous blood samples (5 mL) were collected from all participants before HD. We utilized a chemiluminescent microparticle immunoassay (Innodx, Fujian, China) to measure IgM and IgG antibodies in the plasma of the participants. This assay specifically detects antibodies against the receptor-binding protein of the S1 subunit of the spike protein of SARS-CoV-2 (anti-S1-RBD IgG/IgM). An automatic chemiluminescence instrument (Wan200+; UMIC, Fujian, China) was employed to determine the relative luminescence unit and calculate the signal-to-cutoff ratio (S/CO). According to the manufacturer's guidelines, a sample is deemed nonreactive if the result is less than 1 and reactive if it is equal to or greater than 1. The sensitivity and specificity for IgG were 87.7 % and 99.6 %, respectively, whereas for IgM, they were 92.13 % (95 % confidence interval [CI], 88.15–94.85 %) and 99.15 % (95 % CI, 97.83–99.67 %), respectively.

### 2.4 Ethics statement

This study was approved by the Ethics Committee of The Affiliated Hospital of Hangzhou Normal University on March 13, 2023 (2023 (E2)–KS–018). All procedures were performed in accordance with the 1964 Declaration of Helsinki and its later amendments or comparable ethical standards. All the participants provided written informed consent. This manuscript adheres to the Strengthening the Reporting of Observational Studies in Epidemiology (STROBE) guidelines.

### 2.5 Statistical analysis

Data were evaluated using IBM SPSS Statistics (version 25.0; IBM Corp., Chicago, IL, USA) software, and figures were constructed using GraphPad Prism (version 9.5.0; San Diego, CA, USA). Patient demographic and clinical characteristics are presented as frequencies and percentages for categorical variables and medians (interquartile range, IQR) for continuous variables. The Shapiro–Wilk test was used to assess whether the data were normally or non-normally distributed. Categorical variables were compared using Fisher's exact test. Differences between two independent groups were tested using the Mann–Whitney test or independent *t*-test according to the distribution normality of the variables. The Kruskal–Wallis test was used to compare continuous variables among multiple groups. The Wilcoxon test was used to compare the antibody levels of the paired samples. All tests were two-tailed, and statistical significance was set at p values < 0.05.

## 3 Results

### 3.1 Study design and characteristics of the population

**Fig. 1:**
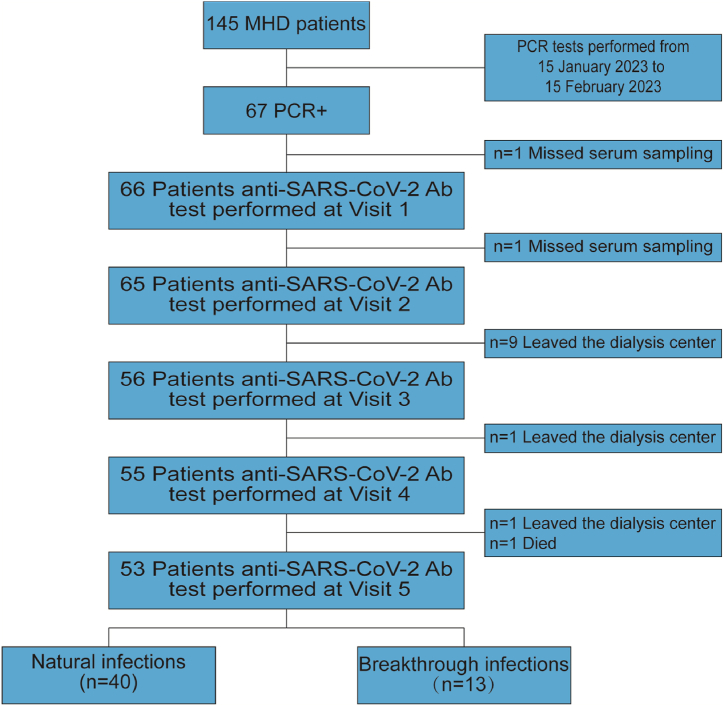
Flowchart of the study. Note: MHD, maintenance hemodialysis; PCR, polymerase chain reaction.

In our HD center, 31 of 145 patients undergoing MHD (21.4 %) were vaccinated with the COVID-19 inactivated vaccine from Sinopharm (Beijing, China), and 114 (78.6 %) were not vaccinated. From January 15, 2023, to February 15, 2023, 67 of 145 (46 %) MHD patients were diagnosed with COVID-19. Two patients were excluded because there was no serum sampling at Visits 1 and 2. Ten patients were excluded because they had left the HD institution at Visits 3 and 4. At Visit 5, two patients were excluded: one for leaving the HD institution and the other who died of natural causes. (Fig. 1).

Fifty-three patients were included and divided into natural (n = 40) and breakthrough infection groups (n = 13). The general characteristics of the participants are presented in Table 1. No significant differences were observed in median age (65 years [IQR, 57–72 years] vs. 66 years [IQR, 57–75 years]), sex (male, 67.5 % vs. 69.2 %; female, 32.5 % vs. 30.8 %), and dialysis vintage (40 months [IQR, 18.2–83.5 months] vs. 19 months [IQR, 9.5–59 months]) between natural and breakthrough infection groups, respectively. All patients in the breakthrough infection group were vaccinated with an attenuated virus vaccine. Among them, 30.8 % (4/13) had been vaccinated with one dose, 30.8 % (4/13) had received two doses, and 38.4 % (5/13) had received three doses (Table 1).

**Table 1:** Demographic and clinical characteristics of the cohort.

	Natural Infections	Breakthrough Infections	*P* value
No.	40	13	
Age, y	65 (57–72)	66 (57–75)	0.839
Sex			
Male (%)	27 (67.5)	9 (69.2)	1.0
Female (%)	13 (32.5)	4 (30.8)	
Dialysis vintage (months)	40 (18.2–83.5)	19 (9.5–59)	0.079
PCR positive onset to Visit 1 (days)	2 (1–14)	2 (1–12)	0.682
PCR positive onset to Visit 2 (days)	29 (27–42)	31 (27–47)	0.373
PCR positive onset to Visit 3 (days)	57 (56–70)	64 (57–72)	0.159
PCR positive onset to Visit 4 (days)	90 (87–100)	88 (87–101)	0.835
PCR positive onset to Visit 5 (days)	120 (118–129)	120 (118–129)	0.648
Vaccine inoculations			
1 dose (%)		4^a^ (30.8)	
2 doses (%)		4^a^ (30.8)	
3 doses (%)		5^a^ (38.4)	
Routine blood parameters after infection (IQR)			
White blood cell count, 10^9^/L	5.5 (4.9–6.9)	5.8 (4.2–7.2)	0.985
Neutrophil count, 10^9^/L	4.1 (3.4–5.2)	4.3 (3.1–4.9)	0.798
Lymphocyte count, 10^9^/L	0.9 (0.6–1.2)	1.0 (0.8–1.2)	0.457
Monocyte count, 10^9^/L	0.49 (0.38–0.59)	0.47 (0.46–0.55)	0.788
Eosinophil count, 10^9^/L	0.1 (0.05–0.23)	0.1 (0.03–0.13)	0.694
Platelet count, 10^9^/L	197 (150–279)	176 (136–220)	0.291
Clinical symptoms			
Fever	20 (50 %)	6 (46.2 %)	0.810
Cough	30 (75 %)	10 (76.9 %)	1.0
Blood oxygen desaturation	7 (17.5 %)	2 (15.4 %)	1.0

IQR, interquartile range; PCR, polymerase chain reaction.

a , Inactivated vaccine from Sinopharm was administered.

### 3.2 Routine blood parameters analysis from natural and breakthrough infection groups

We collected routine blood parameters (median post-disease onset: 2 days) of the natural and breakthrough infection groups using the hospital medical record network system. White blood cell, lymphocyte, neutrophil, monocyte, eosinophil, and platelet counts were not significantly different between the two groups and were all within the normal range (Table 1).

### 3.3 Seropositivity and SARS-CoV-2 RBD antibody response to natural and breakthrough infection groups

**Fig. 2:**
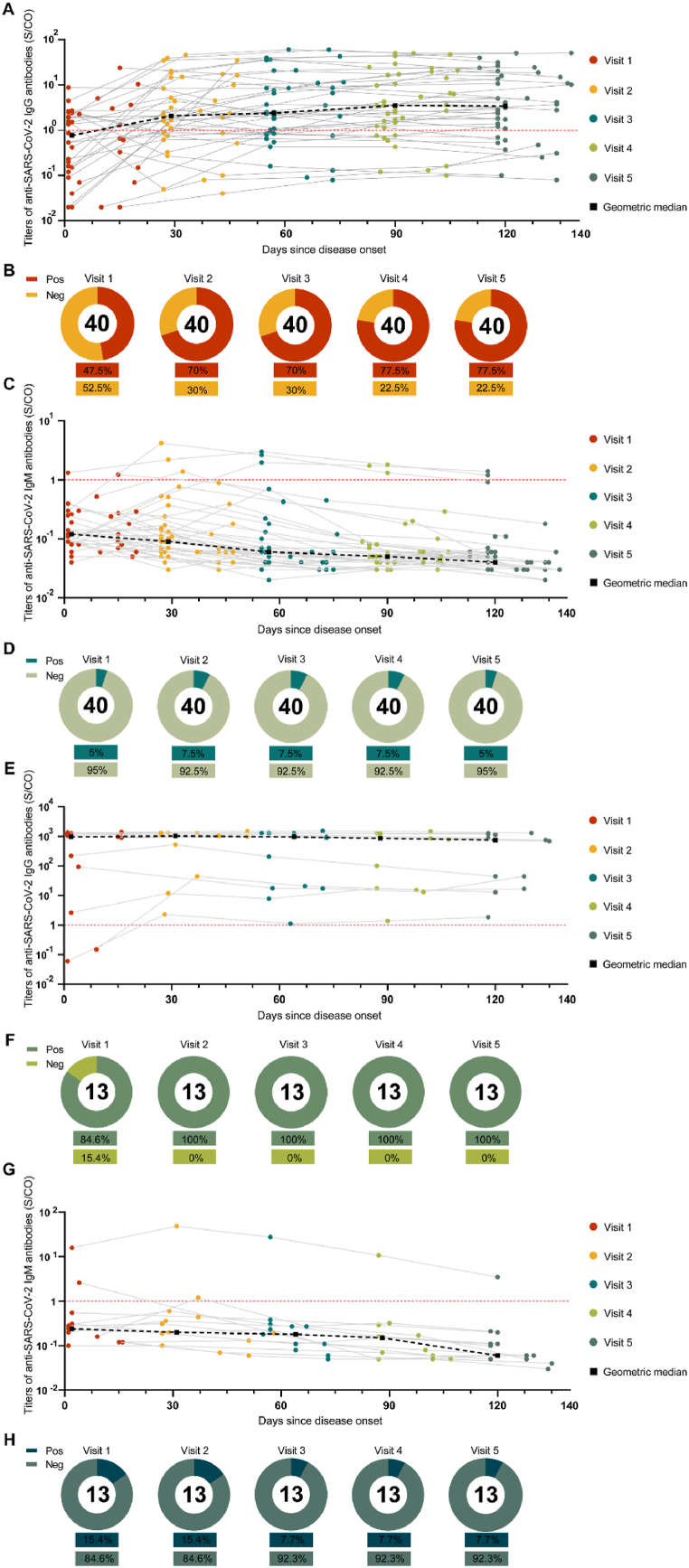
Longitudinal kinetics of SARS-CoV-2 RBD IgM/IgG titers. (A, B, C, and D) SARS-CoV-2 RBD IgG titers, SARS-CoV-2 RBD IgG positivity, SARS-CoV-2 RBD IgM titers, SARS-CoV-2 RBD IgM positivity in the natural infections group during the follow-up period. (E, F, G, and H) SARS-CoV-2 RBD IgG titers, SARS-CoV-2 RBD IgG positivity, SARS-CoV-2 RBD IgM titers, SARS-CoV-2 RBD IgM positivity in the breakthrough infections group during the follow-up period. Dashed lines indicate, Red: RBD cutoff.

**Fig. 3:**
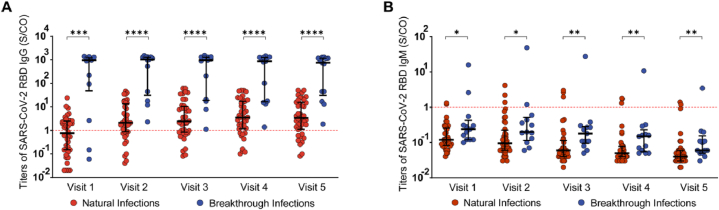
Median SARS-CoV-2 RBD IgG and IgM titers at natural infections and breakthrough infections. Statistical analysis of the difference between IgG (A) and IgM (B) in natural infections and breakthrough infections using the Mann–Whitney test. For all graphs, bars represent the median and interquartile range. Individual patient values are indicated by dots. Dashed lines indicate, Red: RBD cutoff. ∗, p < 0.05; ∗∗, p < 0.01; ∗∗∗, p < 0.001; ∗∗∗∗, p < 0.0001.

SARS-CoV-2 RBD IgG positivity in the natural infection group gradually increased from 47.5 % (19/40) at Visit 1–77.5 % (31/40) at Visit 5. Notably, four individuals in this group did not show detectable SARS-CoV-2 RBD IgG (Fig. 2A and B). The median SARS-CoV-2 RBD IgG titers rose from 0.77 S/CO (IQR, 0.15–2.51) to 3.43 S/CO (IQR, 1.12–15.6) (Fig. 3A). Throughout the follow-up period, SARS-CoV-2 RBD IgM positivity in the natural infection group remained below 10 %, with median titers consistently falling below the designated cutoff values (Fig. 2, Fig. 3B).

SARS-CoV-2 RBD IgG positivity in the breakthrough infection group increased from 84.6 % (11/13) at Visit 1–100 % (13/13) at Visit 2, maintaining a 100 % rate through Visit 5 (Fig. 2E and F). Median IgG titers rose from 957.59 S/CO (IQR, 48.01–1221.66) at Visit 1–1023.3 S/CO (IQR, 31.08–1303.13) at Visit 2, gradually declining thereafter to 751.21 S/CO (IQR, 30.54–1173.63) by Visit 5 (Fig. 3A). Throughout the follow-up period, IgM positivity in the breakthrough infection group decreased from 15.4 % (2/13) at Visit 1–7.7 % (1/13) at Visit 3, with the median IgM titers consistently falling below the designated cut-off values (Fig. 2, Fig. 3B).

We utilized the Mann–Whitney test to examine the differences in SARS-CoV-2 RBD antibodies between the natural and breakthrough infection groups throughout the follow-up period. The findings revealed a significant elevation in SARS-CoV-2 RBD IgG titers within the breakthrough infection group compared with the natural infection group, demonstrating a notable difference (p < 0.001) (Fig. 3A). SARS-CoV-2 RBD IgM titers were significantly higher in the breakthrough infection group than in the natural infections group (p < 0.05). However, the median IgM titers remained below the designated cutoff values (Fig. 3B).

### 3.4 Antibody response to SARS-CoV-2 infection in patients on MHD by age, sex, and vaccination

**Fig. 4:**
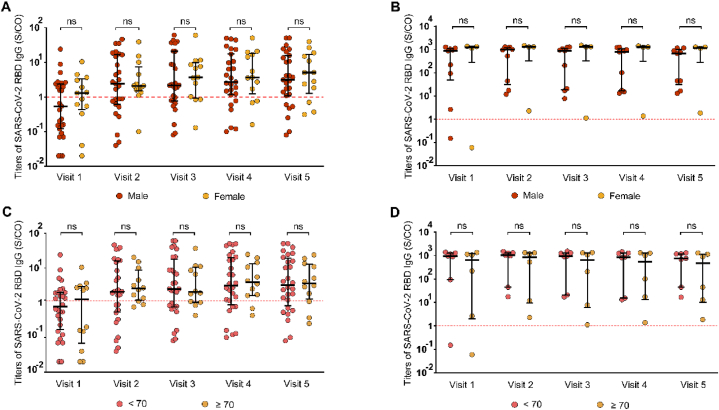
Correlation of SARS-CoV-2 RBD IgG titers with clinical characteristics. SARS-CoV-2 RBD IgG titers in natural and breakthrough infection groups are shown based on (A, B) sex and (C, D) age. Statistical analysis was performed Mann–Whitney test. For all graphs, bars represent the median and interquartile range. Individual patient values are indicated by dots. Dashed lines indicate, Red: RBD cutoff. ns, p > 0.05.

To investigate the potential impact of age and sex on SARS-CoV-2 RBD IgG antibody formation in patients undergoing MHD, we employed the Mann–Whitney *U* test. The analysis revealed no significant differences in SARS-CoV-2 RBD IgG titers between male and female patients (Fig. 4A and B). Additionally, no significant difference was observed in SARS-CoV-2 RBD IgG titers between patients younger than 70 and those 70 years or older (Fig. 4C and D).

**Fig. 5:**
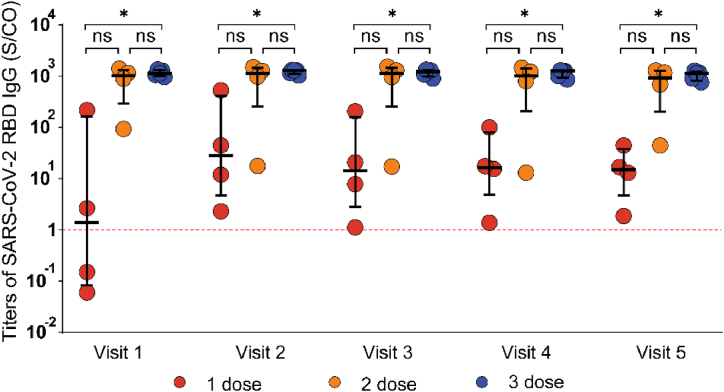
Comparison of SARS-CoV-2 RBD IgG in patients with MHD who received different doses of inactivated vaccines. For all graphs, bars represent the median and interquartile range. Individual patient values are indicated by dots. Dashed lines indicate, Red: RBD cutoff. ∗, p < 0.05.

We used the Kruskal–Wallis test to examine the influence of the number of Sinopharm attenuated vaccine doses on SARS-CoV-2 RBD IgG titers in the breakthrough infection group. Even at Visit 5, the SARS-CoV-2 RBD IgG titers in patients who received three doses were significantly higher than those in patients who received one and two doses of the vaccine (1128.99 S/CO, [IQR, 825.09–1228.94] vs 14.87 S/CO, [IQR, 4.62–37.47] vs 929.11 S/CO, [IQR, 205.16–1267.89]) (p < 0.05). Although no significant difference was observed between the SARS-CoV-2 RBD IgG titers of patients who received two doses and those who received one dose, the titers were significantly higher in patients who received two doses than in those who received only one dose (Fig. 5).

### 3.5 Serum antibody changes using paired data

**Fig. 6:**
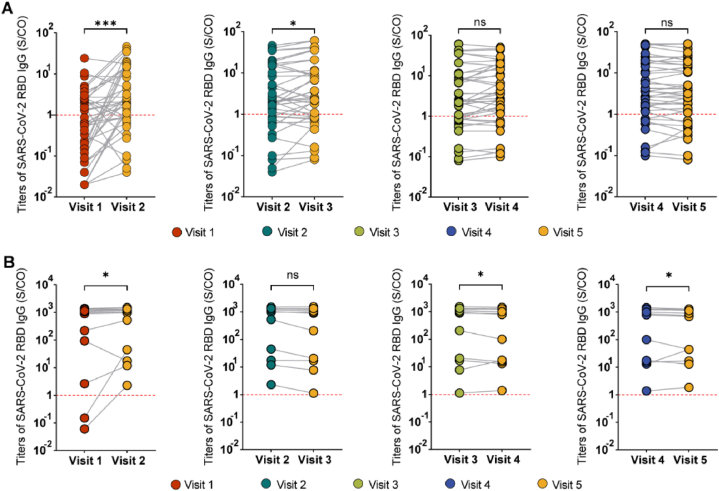
SARS-CoV-2 RBD IgG levels in paired samples at connected follow-up times. A) IgG levels at connected follow-up times were tested using the Wilcoxon test for MHD patients in the natural infection group; B) IgG levels at connected follow-up times were tested using the Wilcoxon test for MHD patients in the breakthrough infection group; Dashed lines indicate, Red: RBD cutoff. ns, p > 0.05 ∗; p < 0.05; ∗∗∗, p < 0.001.

To investigate the pattern of change in SARS-CoV-2 RBD IgG levels more precisely, we paired the data from two consecutive visits and employed the Wilcoxon signed rank sum test. Fig. 6A illustrates that in the natural infection group, SARS-CoV-2 RBD IgG titers increased from 0.77 S/CO (IQR, 0.15–2.51) to 2.1 S/CO (IQR, 0.88–13.53) between Visit 1 and Visit 2 (p = 0.0007). Subsequently, between Visit 2 and Visit 3, SARS-CoV-2 RBD IgG titers increased from 2.1 S/CO (IQR, 0.88–13.53) to 2.41 S/CO (IQR, 0.84–10.47) (p = 0.016). Although SARS-CoV-2 RBD IgG titers were still elevated during the Visit 4 and 5 follow-ups, no significant difference was observed.

In the breakthrough infection group, SARS-CoV-2 RBD IgG titers increased from 957.59 S/CO (IQR, 48.02–1221.66) to 1023.3 S/CO (IQR, 31.08–1303.13) (p = 0.016) between Visit 1 and 2. From Visit 2 to 3, SARS-CoV-2 RBD IgG titers decreased from 1023.3 S/CO (IQR, 31.08–1303.13) to 962.28 S/CO (IQR, 18.96–1299.07) (p = 0.116). During Visits 4 and 5, the SARS-CoV-2 RBD IgG titers gradually decreased to 751.21 S/CO (IQR, 30.54–1173.63) (p = 0.019) (Fig. 6B).

## 4 Discussion

In this study, we describe the SARS-CoV-2 RBD antibody response in patients undergoing MHD (40 natural and 13 breakthrough infections) during a 4-month follow-up period. We showed that SARS-CoV-2 RBD IgM positivity in these patients is extremely low. Even in the acute phase of infection, IgM positivity in natural infections was only 5 % and was slightly higher in patients with breakthrough infections (15.4 %). This is much lower than the IgM positivity of SARS-CoV-2 infection in the general population [[11], [12], [13], [14]]. Therefore, we do not recommend testing for SARS-CoV-2 RBD IgM as an indicator for screening and diagnosing SARS-CoV-2 infection in patients on MHD.

Notably, neutralizing antibodies serve as precise predictors of immune protection. An Israeli study demonstrated an inverse relationship between higher peri-infection neutralizing antibody titers and lower infectivity [15]. However, the complexity of this process limits its practical application in clinical settings. Several studies have established a significant correlation between RBD IgG antibodies and neutralizing antibody titers [16,17]. For instance, Ma et al. reported a 100 % IgG positivity rate 1 month after natural infection in the general population [12]. In another study, IgG levels were measured at 66.5 (36.8–120.3) BAU/mL at 1.7 months post-infection in a naturally infected general population [18]. In contrast, our study indicates that patients on MHD with natural infections exhibit lower IgG titers and positivity rates. These results suggest that patients undergoing MHD may display less robust antibody responses. This is reminiscent of the serological conversion rate of hepatitis B vaccination in patients on HD, which is only 44.3 %. This finding led to the revision of the internationally recognized hepatitis B immunization schedule for patients on HD [19].

Countries have adopted varying strategies regarding vaccination rates and types of vaccines. For instance, Israel and the United States have primarily relied on mRNA vaccines, with data showing that they significantly reduce severe cases and mortality rates. In contrast, China has predominantly used inactivated vaccines [[20], [21], [22]]. While inactivated vaccines have demonstrated some effectiveness in preventing severe illness and death, their efficacy in preventing COVID-19 is lower than in mRNA vaccines [23,24]. This discrepancy became particularly evident after public health policies adjustments, leading to a sharp increase in infection rates [25].

Encouragingly, our findings reveal that SARS-CoV-2 RBD IgG titers and positivity were significantly higher in patients with breakthrough infections than in those with naturally infections. This difference was particularly pronounced in patients who received three vaccine doses, where SARS-CoV-2 RBD IgG levels remained elevated (>800 S/CO), even at 4 months post-infection. Notably, IgG has been associated with a reduced risk of reinfection within a 6-month period, as evidenced by a follow-up study involving 12,541 healthcare workers in the United Kingdom [26]. A recent study further demonstrated that patients on HD with circulating RBD IgG levels below 23 (506 BAU/ml) were at a higher risk of infection than those with RBD IgG levels ≥23. In a retrospective study, none of the patients on HD with anti-S IgG levels exceeding 284 BAU/mL were infected with SARS-CoV-2 [27]. Several studies have highlighted the superiority of mRNA vaccines over inactivated vaccines in terms of success and the induction of neutralizing antibodies [28]. However, it is worth mentioning that in regions or countries where mRNA vaccines are not readily available, inactivated vaccine administration remains a viable option for patients undergoing MHD.

Sex and age did not significantly impact the SARS-CoV-2 RBD IgG antibody response in our study. This contrasts with the findings of Zeng and Carlota Dobaño et al., who suggested a correlation between IgG antibody levels and age [29,30]. The observed differences may arise from variations in the age divisions between studies. In our investigation, the age cutoff was set at 70 years, and the population primarily consisted of middle-aged and older patients undergoing MHD.

This study has several limitations. First, this was a single-center study with a limited number of participants, particularly in the breakthrough infection group, which might have introduced potential biases in the statistical analyses. Second, the absence of pre-infection antibody level data limits our understanding of how antibody levels change before and after infection in patients on MHD with breakthrough infections. Third, the study focused solely on examining the participants’ antibody levels and did not explore cellular immunity and neutralizing antibodies.

Elevated titers of SARS-CoV-2 RBD IgG following SARS-CoV-2 infection in vaccinated patients undergoing MHD compared with their unvaccinated counterparts were observed in this study. Particularly noteworthy were the higher titers found in patients on MHD who received three doses of the inactivated vaccine, with sustained elevated titers of SARS-CoV-2 RBD IgG detected up to the 4 months after infection.

## Funding

This research received no specific grant from any funding agency in the public, commercial, or not-for-profit sectors.

## Data availability statement

Data are available via figshare, https://doi.org/10.6084/m9.figshare.25997671.

## CRediT authorship contribution statement

**Yingji Chen:** Writing – review & editing, Writing – original draft, Data curation, Conceptualization. **Xiaming Zhang:** Investigation, Data curation. **Mi Zhou:** Formal analysis. **Ping Wu:** Methodology, Investigation. **Juzhen Yan:** Supervision, Conceptualization. **Chen Sun:** Project administration, Methodology. **Yinghong Zhang:** Visualization, Formal analysis. **Xiaoyin Zheng:** Project administration, Methodology, Investigation, Data curation.

## Declaration of competing interest

The authors declare that they have no known competing financial interests or personal relationships that could have appeared to influence the work reported in this paper.
